# Fine mapping of QTL and genomic prediction using allele-specific expression SNPs demonstrates that the complex trait of genetic resistance to Marek’s disease is predominantly determined by transcriptional regulation

**DOI:** 10.1186/s12864-015-2016-0

**Published:** 2015-10-19

**Authors:** Hans H. Cheng, Sudeep Perumbakkam, Alexis Black Pyrkosz, John R. Dunn, Andres Legarra, William M. Muir

**Affiliations:** USDA, ARS, Avian Disease and Oncology Laboratory, East Lansing, MI 48823 USA; Microbiology & Molecular Genetics, Michigan State University, East Lansing, MI 48824 USA; INRA, Animal Genetics, GenPhySE, Castanet Tolosan, 31326 France; Department of Animal Sciences, Purdue University, West Lafayette, IN 47907 USA

**Keywords:** Transcriptional regulation, Complex traits, Allele-specific expression, Disease resistance, Genetic architecture, Genomic selection

## Abstract

**Background:**

Marek’s disease (MD) is a lymphoproliferative disease of poultry induced by Marek’s disease virus (MDV), a highly oncogenic alphaherpesvirus. Identifying the underlying genes conferring MD genetic resistance is desired for more efficacious control measures including genomic selection, which requires accurately identified genetic markers throughout the chicken genome.

**Methods:**

Hypothesizing that variants located in transcriptional regulatory regions are the main mechanism underlying this complex trait, a genome-wide association study was conducted by genotyping a ~1,000 bird MD resource population derived from experimental inbred layers with SNPs containing 1,824 previously identified allele-specific expression (ASE) SNPs in response to MDV infection as well as 3,097 random SNPs equally spaced throughout the chicken genome. Based on the calculated associations, genomic predictions were determined for 200 roosters and selected sires had their progeny tested for Marek’s disease incidence.

**Results:**

Our analyses indicate that these ASE SNPs account for more than 83 % of the genetic variance and exhibit nearly all the highest associations. To validate these findings, 200 roosters had their genetic merit predicted from the ASE SNPs only, and the top 30 and bottom 30 ranked roosters were reciprocally mated to random hens. The resulting progeny showed that after only one generation of bidirectional selection, there was a 22 % difference in MD incidence and this approach gave a 125 % increase in accuracy compared to current pedigree-based estimates.

**Conclusions:**

We conclude that variation in transcriptional regulation is the major driving cause for genetic resistance to MD, and ASE SNPs identify the underlying genes and are sufficiently linked to the causative polymorphisms that they can be used for accurate genomic prediction as well as help define the underlying molecular basis. Furthermore, this approach should be applicable to other complex traits.

**Electronic supplementary material:**

The online version of this article (doi:10.1186/s12864-015-2016-0) contains supplementary material, which is available to authorized users.

## Background

Several major issues confront the poultry industry today. With high-density chicken rearing, reduced genetic diversity from industry consolidation [1], and limitations on antibiotic usage, controlling infectious diseases and preventing disease outbreaks are critical for sustaining economic viability, maintaining public confidence in poultry products, and enhancing animal welfare. Among poultry diseases, Marek’s disease (MD), a lymphoproliferative disease caused by the highly oncogenic -herpesvirus Marek's disease virus (MDV), continues to be at or near the top of the list of concerns [2]. Alarm about MD is enhanced by the unpredictable yet recurrent vaccine breaks that result in devastating losses to poultry farms.

The main control strategy for MD is vaccination. However, while these vaccines effectively prevent MD and tumor formation, they do not prevent infection or shedding of pathogenic MDV [3]. And because vaccine viruses and pathogenic MDVs coexistence in MD-vaccinated flocks, it is likely widespread MD vaccination programs have influenced the evolution of pathogenic strains with increasing virulence in the field [4–6]. A recent study [7] has experimentally demonstrated that certain MD vaccinates can select for MDVs that replicate and spread better, which in turn helps to promote viral evolution to higher virulence as this increased viral load favors the chance that one or more cells in a chicken will get transformed.

As a sustainable alternative to vaccination, we have been pursuing a strategy of identifying chickens with enhanced genetic resistance to MD based on genomic selection (GS) for breeding naturally MD resistant flocks [8]. By identifying genetic markers associated with MD resistance genes, it would be possible to select individuals with superior MD genetic resistance rather than using traditional phenotypic selection, which is labor and animal resource intensive involving direct MDV challenge of progeny or siblings to determine estimated breeding values (EBVs). Use of genetic markers offers several advantages including but not limited to (1) improved selection intensity and accuracy, (2) maintenance or integration of new genetic variation into breeding programs, (3) the ability to select birds of either sex at an early age, and (4) obviate the need to expose elite flocks to a hazardous pathogen. The success of this method is contingent on having accurately identified genetic markers.

Identifying underlying causative genes or even tightly linked markers is difficult for genetic resistance to MD, as is the situation for other complex traits. Even with modern tools and efforts [e.g., genome-wide association studies (GWAS)], the significantly-associated genetic markers, typically SNPs, often define linkage disequilibrium (LD) blocks with either no single candidate gene or multiple genes [9]. And while individual genes underlying a portion of the genetic variance for complex traits have been identified in many studies, in general, there are relatively few genes that have a major effect. For those traits that are controlled by large networks of genes where individual genes each have a minor effect, tracing these small signals to identify all the genes in the network is extremely difficult, which partially explains the “missing heritability” problem [10].

There is growing awareness that variation in gene expression is a major factor accounting for phenotypic variations such as those involved in human disease [11]. One technique to identify this variation is to screen for allele-specific expression (ASE). For all genes of interest, the relative expression levels of the two alleles as judged by a marker polymorphism (e.g., SNP) are compared *within* an RNA sample derived from an individual test subject but across biological replicates. If allelic imbalance is observed, then a polymorphic cis-acting element must be present for that gene since allelic variation is by definition reflective of a cis-acting genetic influence. This is in contrast to eQTL analyses where RNA samples *among* individuals, genes with “cis” regulation are defined as being proximal to the gene, but may not be allele specific, which has resulted in many identifications that are actually trans and, thus, incorrectly identify specific genes [12].

The study had two aims. First, we sought to increase our understanding of the complex trait of genetic resistance to MD and use this information to improve commercial poultry breeding via GS. The second goal was to demonstrate the power and utility of using ASE to identify specific genes impacting disease and genetic resistance. Previously, we identified 4528 SNPs in 3718 genes that exhibit ASE in response to MDV infection using F_1_ progeny from experimental inbred White Leghorn layer lines that differ greatly in MD genetic resistance [13, 14]; lines 6 and 7 are MD resistant and susceptible, respectively. Here we show that these genes with cis-regulatory elements account for the majority of the genetic variation between these two experimental White Leghorn (egg laying) lines and verify our genomic predictions by creating lines differentiated for the identified cis-regulatory elements that demonstrate associated changes in MD resistance.

## Results

### Genes with SNPs exhibiting ASE in response to MDV infection are associated with MD genetic resistance

**Fig. 1:**
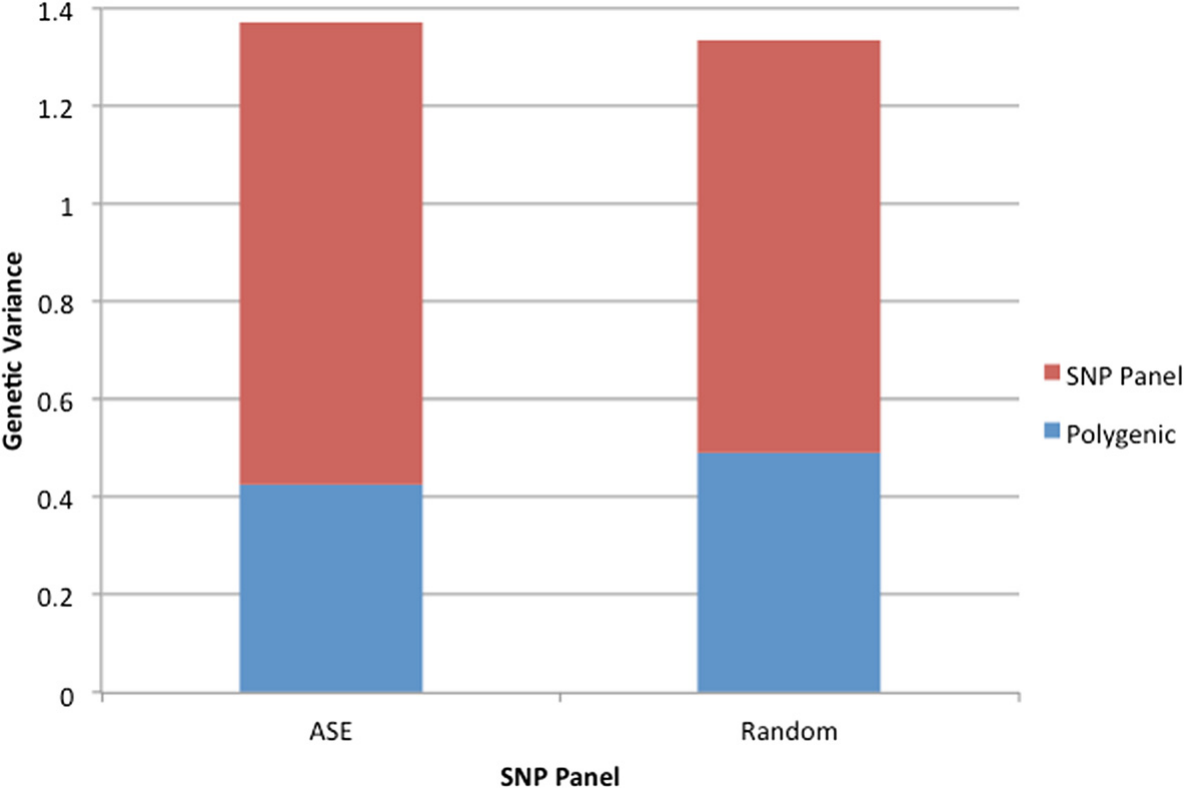
Amount of genetic variation accounted for by the polygenic effect *vs*. each SNP panel. The data was fit using a mixed model containing both a polygenic effect, associated with the pedigree relationship matrix, and a SNP panel, associated with either 1824 ASE SNPs or 3097 random SNPs

**Fig. 2:**
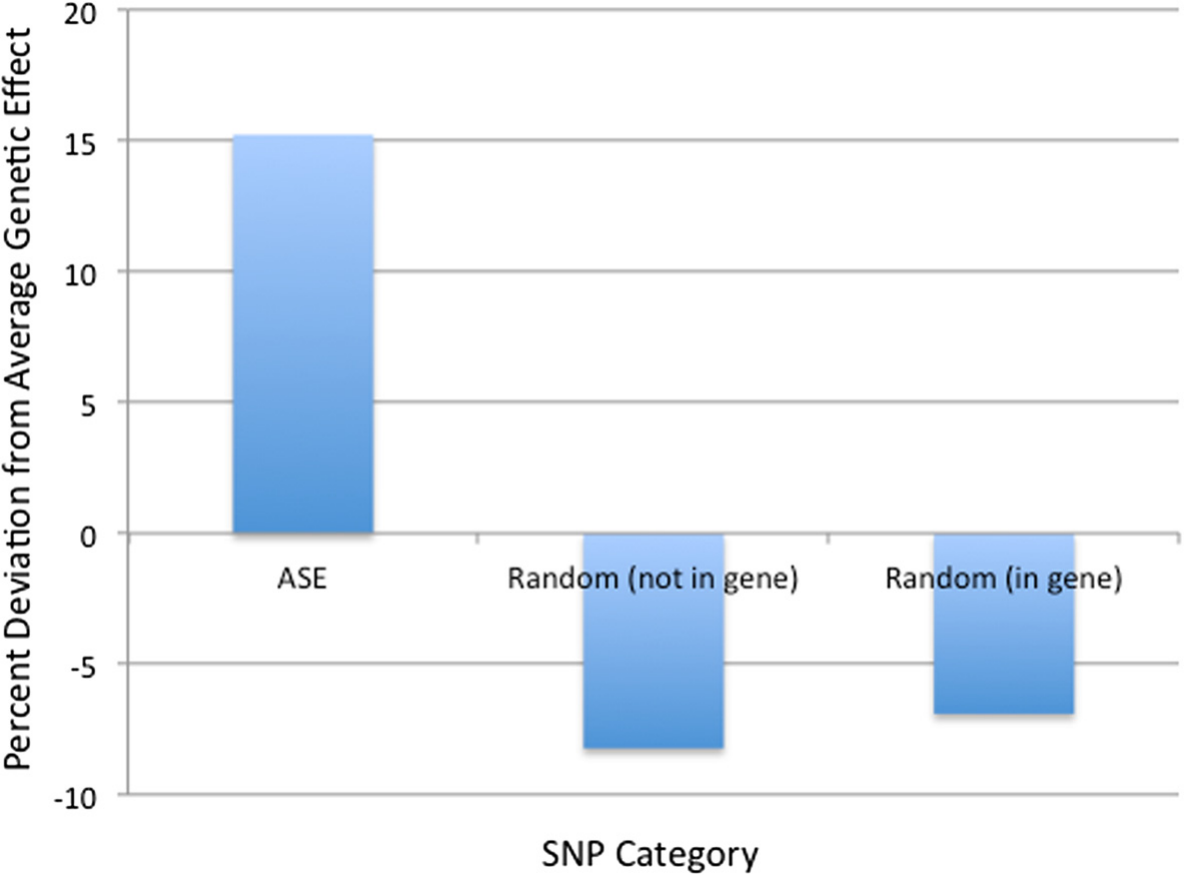
Percent deviation from average genetic effect for each SNP category screened. SNPs used in our genome-wide association analysis (GWAS) were identified as (1) ASE, which showed allele-specific expression (ASE) in response to Marek’s disease virus (MDV) challenge, or (2) were randomly selected based on equally spaced informative genetic markers that were either in genes or between them. The effects associated with the SNPs (a_i_) were estimated from the solutions with all SNPs in the model, then the total contribution of effects to each category, Vg (category), was computed as the sum of the effects in that category squared, which is proportional to the variance explained by SNPs in that category. The relative genetic effects of SNPs in each category (ASE, Random in gene, and Random not in gene) were calculated as 100[Vg(category)-Vg(average)]/Vg(average)

To address whether genes with SNPs exhibiting ASE in response to MDV infection are associated with MD genetic resistance, an advanced intercross (line 6 × 7 F_6_) MD resource population was generated and genotyped with a custom SNP array. To accommodate our 5 K SNP capacity as well as provide genome-wide coverage, the chicken genome was divided into 1 cM blocks based on physical distances determined by the chicken genome assembly [15], then each bin was filled with a suitable ASE or and non-ASE SNPs. In the end, the array included 1824 ASE SNPs plus 3097 “random” SNPs spaced throughout the chicken genome of which 1194 were in genes (Additional file 1: Table S1). Analysis using the GS3 software, a program implementing multiple Bayesian approaches to estimate fixed and random effects, breeding values, and SNP effects on continuous and threshold traits [16], found the selected ASE SNPs account for more than 83 % of the genetic variance in MD resistance. Further analyses comparing effects of ASE SNPs to random SNPs, both within and between coding regions of genes, indicated that on a relative basis, effects associated with ASE SNPs, which are always within coding regions as they are derived from mRNA, account for more of the genetic variance and have less of unaccounted or polygenic component, compared to random SNPs, even though there were ~70 % more random SNPs (Fig. 1). Furthermore, the determined ASE effects were 15.2 % above the average genetic effect, whereas effects for SNPs within and between coding regions of random genes were 6.9 % and 8.2 % below average, respectively (Fig. 2).

**Fig. 3:**
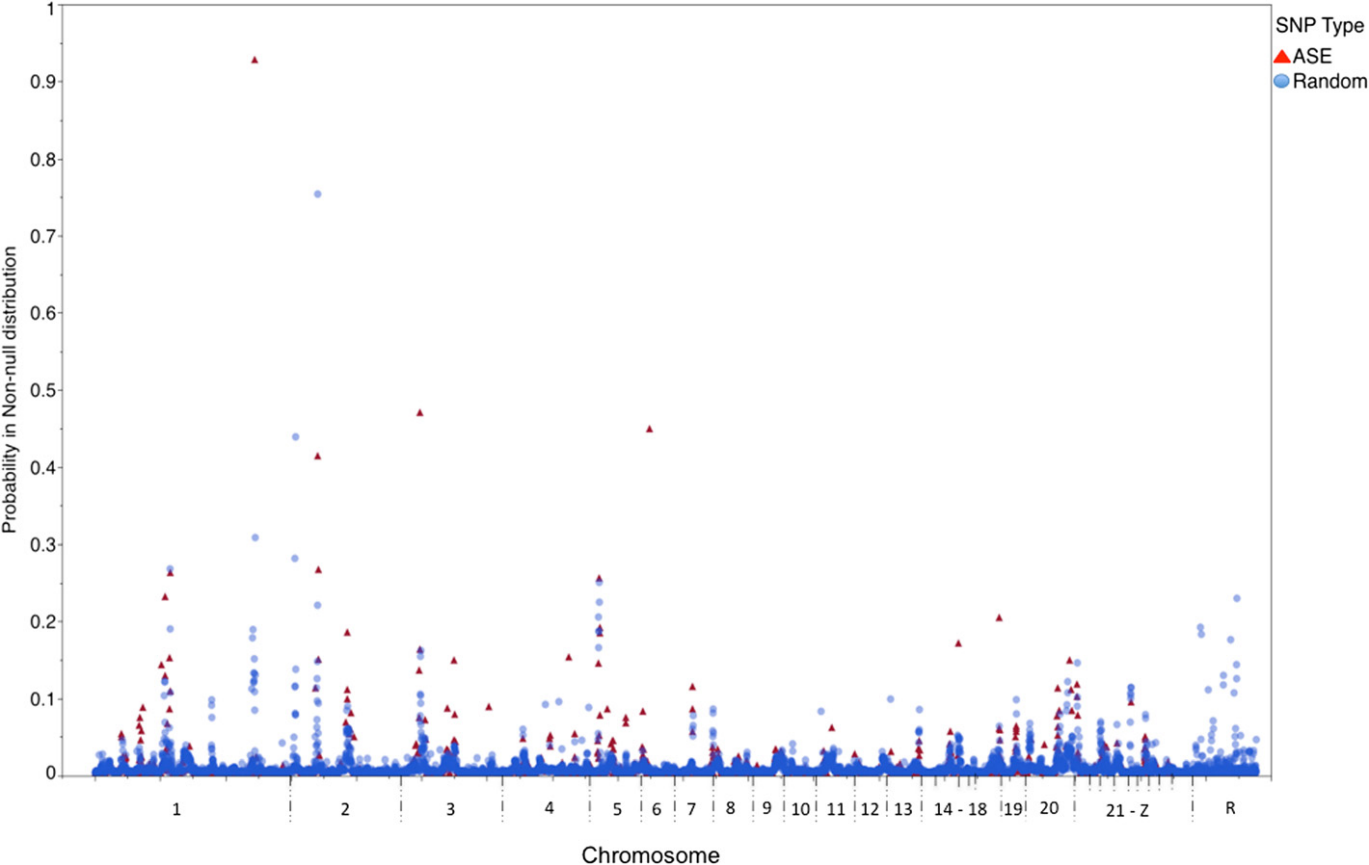
Genome-wide association analysis (GWAS) of Marek’s disease (MD) incidence in experimental layers. The X axis shows the position of each genotyped SNP in the order of the existing chicken genome build with “R” indicating random or unplaced sequences. The probability of each SNP contributing to disease incidence is given on the Y axis. ASE and Random SNPs are denoted by  and , respectively

**Fig. 4:**
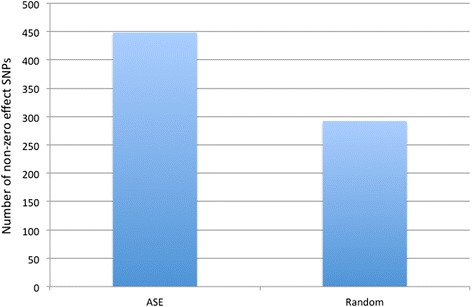
Number of SNPs with non-zero effects. Using BayesCPi, pi is the best estimate of the proportion of the SNPs that have effects. For ASE and random SNPs, pi was 24.6 % and 9.4 %, respectively, which yielded 448 and 292 SNPs, respectively, with non-zero effects

As a consequence, we few exceptions, the ASE SNPs exhibited all the highest genome-wide associations (mixed model; Fig. 3); in the few exceptions where random SNPs were highly associated, many had a nearby significantly-associated ASE SNP. This result was also supported by analyzing the dataset using BayesCPi and estimating pi, which is the best estimate of the proportion of the SNPs that have effects. Pi was found to be 24.6 % and 9.4 % of the ASE and random SNPs, respectively, which resulted in 448 and 292 ASE and random SNPs, respectively, with non-zero effects (Fig. 4). Thus, the ASE approached yielded 156 more SNPs with genetic effects even though there were 1273 less SNPs queried.

### ASE SNPs can accurately predict genetic merit for MD genetic resistance

**Fig. 5:**
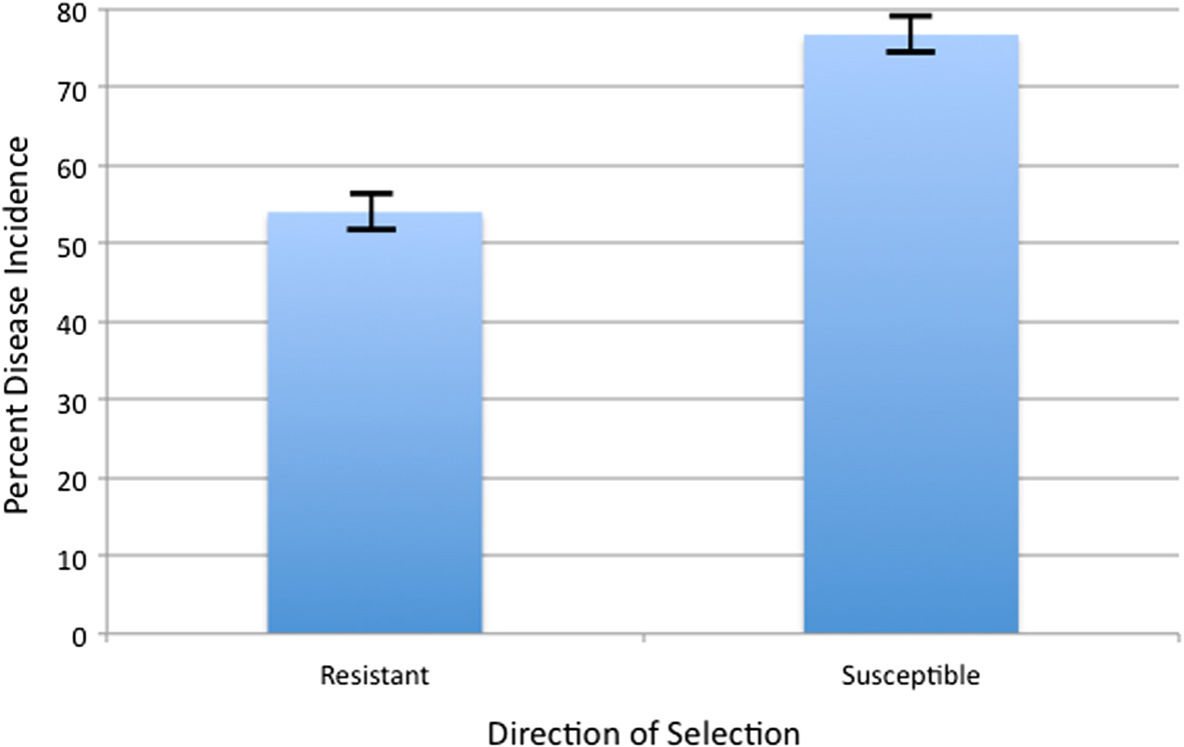
Disease incidence showing response to one generation of selection for genetic resistance or susceptibility to Marek’s disease (MD) based on allele-specific expression (ASE) SNPs only. Genomic estimated breeding values (GEBVs) for 200 F_7_ generation of sires were calculated as the sum of the ASE SNP effects, given the genotype of the sire, and the top 30 and bottom 30 ranked individuals were progeny tested; ~30 progeny per sire. The average incidence and standard deviation for each group of progeny is shown

To validate the association of ASE SNPs with MD genetic resistance, a progeny test was performed. Specifically, 200 F_7_ generation roosters were genotyped, best linear unbiased prediction (BLUP) estimated breeding values (EBVs) based on both SNPs and pedigree calculated, followed by bidirectional selection of roosters based on the SNP EBVs only. The top 30 and bottom 30 ranked roosters were each reciprocally mated to 6 random F_7_ hens, and the resulting ~30 progeny per sire tested for MD incidence; therefore, ~1800 total progeny challenged with MDV and evaluated for MD. As a result, after only one generation of selection, there was a 22 % difference in MD incidence after bidirectional selection based on the ASE SNPs (Fig. 5), which is in line with that predicted based on the genetic variance accounted for by the ASE SNPs and the selection differentials.

**Fig. 6:**
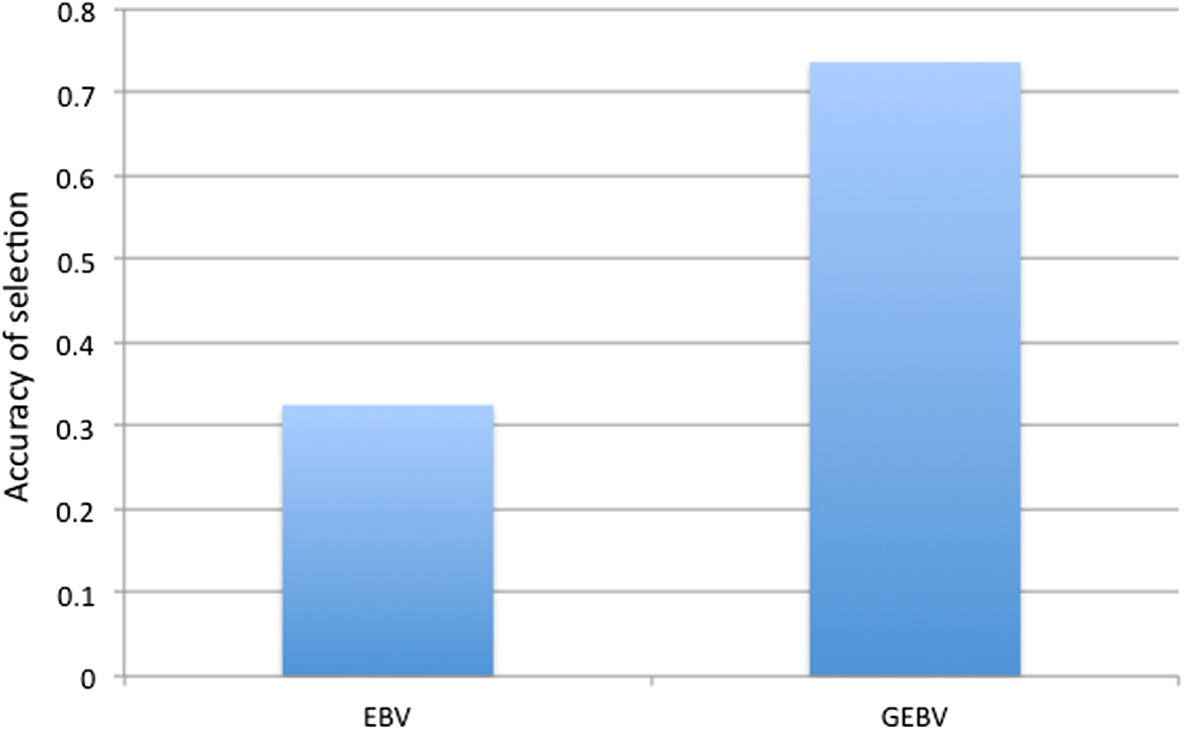
Accuracy of estimated breeding values based on ASE SNPs compared to BLUP. Accuracy was based on a progeny test whereby 60 roosters from the tails of the distributions were each reciprocally mated to 6 random F_7_ hens, and the resulting progeny scored for MD incidence. The accuracy of the breeding values estimated either by pedigree or ASE SNPs was determined by correlating the mean survival of progeny from each rooster with expected breeding values based on either pedigree (EBV) or from SNPS (GEBV)

Based on the progeny test results, the accuracy of selection was compared between BLUP, the current state-of-the-art method that uses pedigree or familial relationships, and genomic selection (GS) based on the ASE SNPs only. Accuracy was calculated as the correlation between the true and predicted estimated breeding values (EBV). For BLUP, the accuracy was 0.325 while the GS EBVs (GEBV) was 0.736 (Fig. 6).

## Discussion

Identifying genes for genetic resistance to MD, like other complex traits, has been challenging. Prior efforts by our group toward this goal have identified three genes and many more candidates by integrating genetic mapping with candidate genes derived through transcript profiling or MDV-chicken protein-protein interactions [8, 17–19]. While these efforts (and more) have aided in our understanding of how genetics and molecular pathways help the bird combat viral infection and transformation, as the majority of the genetic variation is unaccounted for, our knowledge was incomplete. The issue was that these previous strategies mainly relied on linkage between the causative polymorphism and the genetic marker. Unfortunately, due to either the lack of resolution or associations that do not identify specific genes, these approaches were unable to identify or capture the majority of the underlying genes.

As an alternative strategy to alleviate these issues that are confounded by but also rely on LD, we incorporated ASE screens in response to MDV challenge. While ASE does rely on LD, it is limited to the transcriptional unit, which is comprised of the coding gene and accompanying regulatory elements and thus not confounded by output of other linked or unlinked genes. Thus, ASE screens can identify most, if not all, of the genes that have cis-regulatory or genetic elements that control gene expression. Then if differences in gene expression are the major contributor of phenotypic variation, one can identify, map, and determine the contributions of thousands of genes, many of which have small effects, in two steps (ASE SNP identification followed by association analyses) that explain the majority of genetic variation effects for a trait.

It is relevant to highlight the fact that due to the simplicity of this approach, many fewer individuals are required to achieve high power compared to eQTL screens as the latter suffers from the same mapping problems as do other QTL mapping approaches. Another advantage of the ASE approach is to detect candidate genes for further evaluation as the only requirement is, given the proper tissue and timing, that the transcripts be differentially expressed between groups of interest (e.g., uninfected and MDV-infected birds), which is not limited by the effect size. This differs from classic QTL analysis where the effect of the QTL on the trait is directly proportional to the ability to detect it. Furthermore, as ASE is initially based on analysis of RNA within individuals, it is possible that random micro-environmental influences that reduce the heritability of a trait may have less negative influence on the ASE approach because the within individual analysis removes impacts of between individual biological variability and further increases power.

It is also relevant to point out that all analyses in this study were whole-genome based. Effects and *P* values of markers, and phenotype predictions, were estimated simultaneously. This means that our estimates were free from collinearity due to LD among markers. Also it means that we have been able to partition the variance among random SNPs and ASE SNPs, something that cannot be easily done with those GWAS-like approaches that work one marker at a time.

Using this process, we identified genes that account for the majority of genetic resistance to MD, and our predictions were validated by bidirectional selection. To our knowledge, this is the first attempt to quantify the contribution of transcriptional regulation on a whole genome scale. Having said this, recently Gusev et al. [20] reported highly similar results by examining the role of regulatory and coding variants for 11 human diseases. Assuming that DNase I hypersensitive sites found in any human cell are indicative of genomic sites containing regulatory elements, they determined that SNPs in these regions explain an average of 79 % of the heritability; vs. less than 10 % for SNPs in exons. Even though the range of values for the amount of genetic variation accounted for by the putative regulatory elements for the 11 human diseases was large, the fact that nearly the same value on average was declared as determined in our experiment suggests a trend.

Once identified, our results clearly demonstrate that is possible to accurately select for improved genetic resistance to MD using less than 2 K SNPs. We hypothesize that predictions based on ASE SNPs should be more accurate and persist longer over multiple generations of selection, than even whole genome sequencing containing the ASE SNPs as a subset. This is because effects of SNPs that have not recombined, either closely linked or further away, are confounded. Use of distantly related SNPs in the predictions will eventually fail due to recombination in advanced generations. As previously seen with QTL mapping, adding more genotypes does not necessarily increase resolution, only adding biological replicates with different combinations of genotypes is capable of doing that. With an increase in the number of genotyped loci, a corresponding increase in biological replicates is necessary to properly train the model. For example, given a simply inherited trait based on a single QTN and two data sets, one with 3 phenotypes associated with genotypes at the causative QTN, and another data set with the complete genome sequence of each individual. Both data sets include the true QTN while the latter also includes noise. The noise will decrease the accuracy of prediction in the first generation while in the latter generations become even worse because distantly linked loci will recombine and become less predictive. The parallel to complex traits and high density genotyping is similar.

Conceptually a SNP chip with only the causative QTN would be the most accurate with predictions that would not decay over generations due to recombination and/or imperfect training, but would still loose predictive ability due to fixation of the favorable alleles [21]. An ASE SNP chip comes closer to that ideal chip than a random set of SNPs regardless of how dense. From a practical aspect, this means that existing SNP arrays can be easily enhanced by including “add-on” content for the ASE SNPs.

Examination of the top SNPs was indicative of the power of this approach. There were 13 SNPs with *P* (non-null) values greater than 0.25 of which 7 were ASE. And for 2 of the top 6 random SNPs, there were ASE SNPs that tagged the same gene. For example, both a random SNP at chr. 2, position 40,419,282 (ranked as the second highest) and an ASE SNP at chr. 40, position 442,578 (sixth highest) identified CKLF-like marvel transmembrane domain-containing 7 (CMTM7). Most of the candidate genes for these top hits were associated with cell cycle regulation, metabolism, and tumor biology, which is consistent with our prior QTL results that suggested the underlying genes were either involved in restriction of viral replication or, more often, control of neoplastic transformation [22].

Although satisfying, the ultimate goal is to identify the causative polymorphisms to connect phenotype with genotype, which theoretically should allow for near perfect predictions. ASE is most likely the result of different allelic rates of transcription, transcript processing, or transcript stability. As ASE SNPs are found only in coding regions as they rely on screening mRNAs, identifying causative variants requires knowledge of transcriptional regulatory regions for each gene. With regard to investigating polymorphisms in promoters of candidate genes, we have had some success in profiling for binding sites of the MDV Meq, the viral oncogene and a bZIP transcription factor, that result in ASE in response to viral infection [23, 24]. Similar levels of success have been obtained by screening for alternative splicing under control and MDV-infected conditions [25]. The limited success suggests that it may be more productive to examine polymorphisms that affect transcription factor binding in enhancers as they are thought to be the major contributors for expression and trait variation [26].

## Conclusions

We conclude that ASE based SNPs are functionally linked to causative polymorphisms that alter transcriptional levels in genes manifesting changes due to disease incidence. Our results also clearly show that variation in cis-regulatory elements is the major mechanism that accounts for the majority of variation in MD genetic resistance between these two experimental lines, which supports the hypothesis that phenotypic variation in traits is mainly due to changes in regulation of gene expression rather than protein composition. Our results also suggest that complex traits are controlled by many genes, most of which have small effects. And in theory, this method should be generally applicable to other infectious diseases and complex traits at the whole genome level or to fine map existing QTL or associations.

## Methods

### Bird populations and phenotypic measurements for Marek’s disease

The highly inbred Avian Disease and Oncology Laboratory (ADOL) line 6 (MD resistant) and line 7 (MD susceptible) experimental White Leghorn chickens were intermated to produced F_6_ and F_7_ birds. To test disease susceptibility, at 2 days of age, each bird was injected intra-abdominally with 2000 pfu MDV (JM strain) and housed for up to 8 weeks in Horsfall-Bauer isolators. Moribund birds, or those that survived up to eight weeks post challenge, were terminated and examined via necropsy. Birds were scored as having MD if they displayed visceral tumors or had enlarged nerves. All experiments were approved by the USDA, ADOL Animal Care and Use Committee (ACUC). The ACUC guidelines established and approved by the ADOL ACUC (April 2005) and the Guide for the Care and Use of Laboratory Animals by the Institute for Laboratory Animal Research (2011) were followed throughout the experiments.

### Design of custom SNP array

To achieve genome-wide coverage within a limit of 5 K SNPs, the ~ 1 billion bases of the chicken genome was partitioned into ~3000 1 cM bins based on the derived physical distances per chromosome from the chicken genome assembly [15]. Priority was given first to the 4528 previously identified ASE SNPs [14]. This class of genetic markers was screened using the Axiom myDesign Array, Affymetrix (Santa Clara, CA, USA), and those that were scored as recommended were retained. For bins lacking a genetic marker, SNPs based on the genomic sequences of lines 6 and 7 (unpublished) in the desired regions were similarly screened and retained. The final array composition was finalized by selecting 1 or 2 SNPs per bin with the maximum content being up to 5 K SNPs (see Additional file 1: Table S1).

### Association analysis

Markers were fit simultaneously using a BayesCPi model, which produced estimates of marker effects and posterior probabilities of markers having an effect different from zero [27, 28]. To estimate QTL effects associated with those ASE loci, over 1000 pullets from a 6 × 7 F_6_ MD resource population were produced, challenged with MDV as described above, and scored as either disease present or absent based on necropsy. DNA was isolated from 10 μl of blood from each bird using 96-Well Plate Blood Genomic DNA Mini-Preps kits (BioPioneer Inc., San Diego, CA, USA) then genotyped by DNA Landmarks (Saint-Jean-sur-Richelieu, Quebec, Canada) using the custom 5 K SNP array described above. The data were subjected to mixed model analysis using the binary option of the GS3 set of programs [16] with SNPs and/or pedigree effects treated as random. The binary option estimates SNP effects and variance components on the underlying liability scale.

### Accuracy of selection analyses

Roosters in the F_7_ generation were genotyped, BLUP EBVs based on SNPs and pedigree were calculated, and were bidirectionally selected based on the SNP EBVs. The top 30 and bottom 30 ranked roosters were each mated to 6 random F_7_ hens, and ~30 progeny per sire tested for MD resistance over a total of 3 hatches. The accuracy of selection was determined from the correlation of EBVs estimated based on either SNPs or pedigree with progeny test performance.

### Disclaimer

Mention of trade names or commercial products in this publication is solely for the purpose of providing specific information and does not imply recommendation or endorsement by the U.S. Department of Agriculture.

## Additional files

Additional file 1: Table S1. Content of custom SNP array. (XLSX 424 kb)
